# Genetic and Molecular Functional Characterization of Variants within *TNFSF13B*, a Positional Candidate Preeclampsia Susceptibility Gene on 13q

**DOI:** 10.1371/journal.pone.0012993

**Published:** 2010-09-29

**Authors:** Mona H. Fenstad, Matthew P. Johnson, Linda T. Roten, Per A. Aas, Siri Forsmo, Kjetil Klepper, Christine E. East, Lawrence J. Abraham, John Blangero, Shaun P. Brennecke, Rigmor Austgulen, Eric K. Moses

**Affiliations:** 1 Department of Cancer Research and Molecular Medicine, Norwegian University of Science and Technology, Trondheim, Norway; 2 Department of Genetics, Southwest Foundation for Biomedical Research, San Antonio, Texas, United States of America; 3 Department of Public Health and General Practice, Norwegian University of Science and Technology, Trondheim, Norway; 4 Department of Perinatal Medicine/Department of Obstetrics and Gynaecology, Royal Women's Hospital and University of Melbourne, Parkville, Australia; 5 The School of Biomedical Biomolecular and Chemical Sciences, The University of Western Australia Crawley, Perth, Australia; Innsbruck Medical University, Austria

## Abstract

**Background:**

Preeclampsia is a serious pregnancy complication, demonstrating a complex pattern of inheritance. The elucidation of genetic liability to preeclampsia remains a major challenge in obstetric medicine. We have adopted a positional cloning approach to identify maternal genetic components, with linkages previously demonstrated to chromosomes 2q, 5q and 13q in an Australian/New Zealand familial cohort. The current study aimed to identify potential functional and structural variants in the positional candidate gene *TNFSF13B* under the 13q linkage peak and assess their association status with maternal preeclampsia genetic susceptibility.

**Methodology/Principal Findings:**

The proximal promoter and coding regions of the positional candidate gene *TNFSF13B* residing within the 13q linkage region was sequenced using 48 proband or founder individuals from Australian/New Zealand families. Ten sequence variants (nine SNPs and one single base insertion) were identified and seven SNPs were successfully genotyped in the total Australian/New Zealand family cohort (74 families/480 individuals). Borderline association to preeclampsia (p = 0.0153) was observed for three rare SNPs (rs16972194, rs16972197 and rs56124946) in strong linkage disequilibrium with each other. Functional evaluation by electrophoretic mobility shift assays showed differential nuclear factor binding to the minor allele of the rs16972194 SNP, residing upstream of the translation start site, making this a putative functional variant. The observed genetic associations were not replicated in a Norwegian case/control cohort (The Nord-Trøndelag Health Study (HUNT2), 851 preeclamptic and 1,440 non-preeclamptic women).

**Conclusion/Significance:**

TNFSF13B has previously been suggested to contribute to the normal immunological adaption crucial for a successful pregnancy. Our observations support *TNFSF13B* as a potential novel preeclampsia susceptibility gene. We discuss a possible role for *TNFSF13B* in preeclampsia pathogenesis, and propose the rs16972194 variant as a candidate for further functional evaluation.

## Introduction

Preeclampsia is a major cause of fetal and maternal morbidity and mortality in pregnancy, with an incidence ranging from 2–5% [1]. A complete understanding of the etiology and pathogenesis of the preeclampsia syndrome remains elusive. The clinical manifestations of hypertension and proteinuria usually emerge after 20 weeks of pregnancy, and are caused by inflammatory changes and endothelial dysfunction [2], [3]. Impaired placentation in the earlier stages of pregnancy is an underlying pathological feature [4]. However, immunological changes occurring before placentation [5] and even before implantation [6] are also implied in the pathogenesis. Therefore a three stage model for preeclampsia is proposed [7], [8] in which immunological dysfunction (stage 1) is followed by impaired placentation (stage 2), leading to an enhanced inflammatory state and overt preeclampsia (stage 3). Maternal-fetal immune maladaption is an intriguing aspect of preeclampsia pathogenesis, for which there is both epidemiological and biological evidence [9]–[14]. Importantly, the theory implies a mechanism which by partial failure will lead to poor placentation, but by more severe failure will cause spontaneous abortion. Indeed, observations of immunological pathogenic factors place preeclampsia as an intermediate phenotype between miscarriage and successful pregnancy [8].

Like in the majority of other common complex disorders, the mode of preeclampsia inheritance is unclear [15]–[17]. By examining the probability of co-segregating loci within familial cohorts, several loci most likely to harbor maternal susceptibility genes have been identified [18]–[24]. Genome-wide linkage studies in our Australian/New Zealand (Aust/NZ) familial cohort initially identified a maternal preeclampsia susceptibility locus to chromosome 2q [23], [25]. Re-analysis of the Aust/NZ data set, assuming an underlying inherent quantitative liability for preeclampsia, resolved and strengthened the chromosome 2 linkage signal to 2q22 [24]. Two additional novel maternal preeclampsia susceptibility quantitative trait loci (QTLs) on chromosomes 5q and 13q were revealed [20], [24]. An extended Aust/NZ familial cohort and an independent retrospectively ascertained Norwegian case/control cohort (the HUNT2 cohort) have been utilized to identify maternal preeclampsia susceptibility genes at these QTLs. Association to the activin A receptor, type IIA (*ACVR2A*) [26], [27] and the endoplasmic reticulum aminopeptidase 2 (*ERAP2*) [28] genes at the 2q22 and 5q QTLs, respectively, has been reported. Priorization of candidate susceptibility genes at the 13q QTL, was performed using the database text-mining program GeneSniffer (www.genesniffer.org) [20], [24], [28], literature searches and interrogating publically available SNP loci in the Aust/NZ and Norwegian cohorts (NCBI SNP database, dbSNP build 125, Sep 2005) [29]. This preliminary assessment identified the tumor necrosis factor (ligand) superfamily 13B (*TNFSF13B*) as our most promising candidate gene [29].

TNFSF13B, also known as BAFF, BLYS, TALL-1, zTNF4, THANK, CD257, TNFSF20 and DTL, is a member of the TNF superfamily. This protein is active both as a membrane-bound and soluble ligand. Originally discovered as an important stimulator of B-cell proliferation and immunoglobulin production [30], [31], TNFSF13B has later been shown to hold various roles in the innate immune system [32]. Both malignant [33]–[35] and autoimmune [36], [37] B-cell diseases have been linked to this protein. Furthermore, TNFSF13B has been implicated in normal placental development [38], [39], with reduced expression in recurrent spontaneous miscarriage patients [40].

The current study aimed to identify potential functional and structural variants in *TNFSF13B* by re-sequencing the proximal promoter area and coding regions of the gene in preeclamptic individuals from our Aust/NZ families. Identified variants were tested for association with maternal preeclampsia genetic susceptibility in the extended Aust/NZ families. Associated variants were further assessed by formal molecular genetics analyses followed by attempts to independently replicate genetic association findings in a large Norwegian case/control cohort.

## Materials and Methods

### Ethics

#### Australia

Ethical approval for the recruitment of Aust/NZ preeclampsia family members was granted by the Royal Women's Hospital Research and Ethics Committees, Melbourne, Australia. Written informed consent was obtained from study participants prior to them being phlebotomized. Ethical approval for the molecular genetic investigation across the 13q QTL in The 74 Family Cohort was obtained from The University of Texas Health Science Center at San Antonio, Institutional Review Board. Data were analyzed anonymously.

#### Norway

Prior approval to link the information in the HUNT and MBRN databases, to use the Norwegian case/control cohort for genetic studies, and to export samples was obtained by the Regional Committee for Medical Research Ethics, Norway and approved by the National Data Inspectorate and The Directorate of Health and Social Welfare. Ethical approval for genotyping and statistical analysis of the Norwegian case/control cohort was also obtained from The University of Texas Health Science Center at San Antonio, Institutional Review Board. Data were analyzed anonymously.

### Aust/NZ Study Population

The Aust/NZ familial cohort consists of the original set of 34 (26 Australian and eight New Zealand) families that we have previously used to localize the 2q, 5q and 13q preeclampsia susceptibility QTLs and an additional 40 (Australian) preeclampsia families that we have subsequently ascertained and recently described [26]. The entire familial sample is herein called “The 74 Family Cohort”. All family members are of Caucasian origin. Preeclampsia diagnosis in the Aust/NZ study population was performed by qualified clinicians, using criteria set by the Australasian Society for the Study of Hypertension in Pregnancy (new onset proteinuria, ≥0.3 g/d and either an increase from baseline blood pressure of 15/25 mmHg or absolute values ≥140/90 mmHg on at least two occasions 6 h or more apart) [41], [42] as described in detail elsewhere [23], [26]. Women who met the preeclamptic criteria of new onset of hypertension and proteinuria in pregnancy, and experienced convulsions or unconsciousness in the prenatal period were classified as having had eclampsia. Women with pre-existing hypertension or other medical conditions known to predispose for preeclampsia (e.g. renal disease, diabetes, twin pregnancies or fetal chromosomal abnormalities) were excluded. Family members were coded as 1) affected, 2) unaffected or 3) unknown (e.g. male, non-fertile women).

### Norwegian Study Population

All women in the Norwegian cohort were identified from Nord-Trøndelag County in Norway as part of a large multipurpose health survey conducted during 1995–1997 (the Nord-Trøndelag Health Study, HUNT2) [43]. Preeclamptic women and women who had non-preeclamptic pregnancies were retrospectively identified in the HUNT cohort by linking the HUNT database to the database at the Medical Birth Registry of Norway (MBRN) as previously described [44], [45]. Preeclampsia was defined in accordance with the National High Blood Pressure Education Program Working Group on High Blood Pressure in Pregnancy (new onset hypertension, blood pressure ≥140/90 mmHg, and proteinuria, ≥0.3 g/d in pregnancy) [46] using diagnosis codes ICD-8 (before 1998) and ICD-10 (after 1998) as previously described [44], [45]. Preeclamptic women with multiple pregnancies were excluded. Two controls per case were identified at random among parous women in the HUNT2 cohort with no registered preeclamptic pregnancy in the MBRN. Information stored in hospital records was retrospectively examined by an independent obstetrician for validation of the diagnosis reported to MBRN.

### Positional Candidate Gene Sequencing

Two kb of the proximal promoter (upstream of the translation start site) and all six exons (translated or untranslated) of the 13q preeclampsia QTL candidate gene, *TNFSF13B* (NM_006573.3) were sequenced in 48 preeclamptic women. These women are a selection of founders or probands chosen from the most informative pedigrees in The 74 Family Cohort. This sequencing sample set will give a greater than 99% probability of detecting any polymorphism that has a frequency of 0.05 or greater.

### Primer Design for *TNFSF13B* Gene Sequencing

Sequence information for use as a reference template was obtained from the UCSC Genome Browser (Human, Mar. 2006 [NCBI/hg 18]). Sequencing primers were designed using Primer-BLAST (http://www.ncbi.nlm.nih.gov/tools/primer-blast). Primers were designed to be between 20 and 27 bp in length with an annealing temperature between 55°C and 63°C and within 1°C of each other (Table 1).

**Table 1:** Primers used for *TNFSF13B* PCR amplification and sequencing.

Name	Primer Sequence	Fragment size (bp)	Annealing temperature (°C)
Promoter 1 F	AGACGTTACAAGCACAGTTGTAGAA	652	60
Promoter 1 R	CCGAGCAGTGTACACATTGAA		60
Promoter 2 F	CATAGGAATGATCTAATGGACTTTAG	631	57
Promoter 2 R	CATTCTAGTCCTGCCTTATCCT		57
Promoter 3 F	TTCTCCACTTTGCACTATATCATTTC	585	58
Promoter 3 R	AACATGCATAAACTTTTTCCTTCTG		58
Promoter 4 F	TAGTATCATATTGAGCGGGGACTTA	728	58
Promoter 4 R	CTTTCTGCATCTCTACCCCTACTG		58
Exon 1 F	TAAGGGGTTTTAAATCTACTTGAGCAT	664	60
Exon 1 R	TGCAAACTCACTTTCAGTCCC		60
Exon 2 F	TCACGGTGGTGTCTTTCTACC	661	62
Exon 2 R	GCATTATCTACCTGAGGAAACACATA		62
Exon 3 F	AATGTCATGCAATCAATGTAAAAAGT	639	57
Exon 3 R	TCTAAGTGGAAAAAGTACTGGGGATA		57
Exon 4/5 F	GAGGTAGCTTAACAACTAAATGGAGG	559	60
Exon 4/5 R	TTGAGGAATGTCTTTCTGTCTATTTG		60
Exon 6 F	AGATAATTGCAATGGTTTAGAAGTCC	430	58
Exon 6 R	TAGTTTCAGCAAACCAAAACAAATAG		58
Exon 6 seq F	TTTATTTAAGATTCTTTTCTTTTCTGTTG	261	
Exon 6 seq R	TTGGTATTTTCAGTTAGATTCTTTCTT		

F; Forward primer, R; Reverse primer. For exon 6 an extra set of sequencing primers internal to the PCR amplicon of 430 bp was used.

### *TNFSF13B* Gene Sequencing

Extraction of genomic DNA from peripheral blood samples has been previously described [23]. PCR was performed with 20 ng genomic DNA in a 5 µl reaction containing 0.25 U HotStarTaq DNA Polymerase (QIAGEN), 1× QIAGEN PCR buffer, 0.2 mM dNTP, and 0.2 mM of each forward and reverse primer (Table 1). A GeneAmp 9700 thermal cycler (Applied Biosystems) was used for PCR amplification. After an initial denaturation step at 95°C for 15 min, 40 cycles of 94°C for 30 s, a primer pair specific annealing temperature (Table 1) for 30 s, and 72°C for 30 s were run followed by a final extension step of 72°C for 10 min. PCR products were purified using ExoSAP-IT (Amersham Biosciences) according to the manufactures instructions before they were used as a template for sequencing. Sequencing reactions were performed independently for both sense and anti-sense DNA strands using 1 µl purified PCR product in a 5 µl reaction, containing 0.25 µl AB BigDye Terminators v3.1 (Applied Biosystems), 1× AB BigDye Terminator v3.1 buffer (Applied Biosystems) and 1.6 mM of either forward or reverse primer. Sequence reaction amplification was performed on a GeneAmp 9700 thermal cycler using standard cycling conditions, 96°C for 1 min followed by 25 cycles of 96°C for 10 s, 50°C for 10 s and then 60°C for 4 min. The Applied Biosystems BigDye XTerminator purification kit was used according to manufacturer's instructions to purify all sequenced products. Purified sequence reactions were electrophoretically separated on an Applied Biosystems 3730xl DNA Analyzer. Sequence variant identification was performed using Applied Biosystems' SeqScape software v2.6.

### SNP Genotyping in the Aust/NZ Study Population

All *TNFSF13B* SNPs identified in our sequencing experiments were incorporated into a custom Illumina SNP pool and genotyped back in The 74 Family Cohort. Briefly, SNP designs were uploaded to Illumina's Assay Design Tool to design a custom GoldenGate SNP pool with VeraCode technology (Illumina Inc., CA). The design of two allele specific oligos and one locus specific oligo in conjunction with a universal set of amplification primers followed by hybridization to complementary VeraCode bead types makes the GoldenGate assay with VeraCode technology highly robust and specific in a small to medium multiplex reaction. Each VeraCode microtitre bead plate was imaged on the Illumina BeadXpress Reader System using Illumina VeraScan image data acquisition software (version 1.1.9.2). SNP genotype clustering and individual sample genotype calls were interrogated using the Illumina GenomeStudio software, Genotyping Module (version 1.1.9). As an added measure we confirmed genotype calls made by GenomeStudio against the sequence data obtained from our sequencing sub-set of The 74 Family Cohort (n = 48).

### Bioinformatic Evaluation of SNPs

To predict possible functional relevance of the detected *TNFSF13B* variants, we used different publicly available bioinformatic tools for identifying transcription factor binding sites in DNA sequences (http://www.gene-regulation.com), as well as Transfac® Professional and MotifScanner. The programs use different approaches to utilize the library of mononucleotide weight matrices in the TRANSFAC® [47] and Jaspar [48] databases.

### Electrophoretic Mobility Shift Assays (EMSA)

HeLa and T47D total nuclear protein extract was prepared and stored as described [49]. Total protein was determined using the Bio-Rad Protein Assay Reagent. The DNA oligonucleotides (0.025 µmol) (Sigma-Aldrich) used to assay the three *TNFSF13B* variants are presented in Table 2. All oligonucleotides were 5′ end-labeled using T4 polynucleotide kinase (PNK) (New England Biolabs) and [γ33P] ATP (3000 Ci/mmol) (PerkinElmer) and annealed to their complementary unlabeled oligonucleotides as previously described [49]. The samples were purified according to manufacturers' instructions through G25 Microspin™ columns (GE Healthcare). The EMSA reactions were carried out in binding buffer (4% glycerol, 1 mM MgCl2, 0.5 mM EDTA, 0.5 mM DTT, 50 mM NaCl, 10 mM Tris–HCl (pH 7.5), 0.25 mg/ml poly(dI-dC)) in a final volume of 10 µl. Nuclear extract (7 µg) was incubated with double-stranded competitor oligonucleotides for 30 min at room temperature, followed by the addition of 50 fmol of P33 labeled oligonucleotide and then incubated for another 30 min. Samples were mixed with 1 µl of 10× loading buffer (250 mM Tris–HCl (pH 7.5), 0.1% bromophenol blue, 40% glycerol) and run on a 4% polyacrylamide gel (37.5∶1 acrylamide:bisacrylamide, 2.5% glycerol, 0.5× TBE) at 300 V. The gels were fixed in 50% ethanol and 10% acetic acid for 1 h followed by Phosphor Imager analysis (Bas-1800II) (Fujifilm) of the dried gel.

**Table 2:** Oligonucleotides used for Electrophoretic mobility shift assays.

SNP	Allele	F/R*	Sequence
rs16972197	G	F	5′-GCTTTCCCTTGACTGTGCCAATCC-3′
	G	R	5′-GGATTGGCACAGTCAAGGGAAAGC-3′
	C	F	5′-GCTTTCCCTTCACTGTGCCAATCC-3′
	C	R	5′-GGATTGGCACAGTGAAGGGAAAGC-3′
rs16972194	G	F	5′-AAACTTCTTACTTAAGACTGTGTGGAAATGTAGAGT-3′
	G	R	5′-ACTCTACATTTCCACACAGTCTTAAGTAAGAAGTTT-3′
	A	F	5′-AAACTTCTTACTTAAGACTGTATGGAAATGTAGAGT-3′
	A	R	5′-ACTCTACATTTCCATACAGTCTTAAGTAAGAAGTTT-3′
rs56124946	C	F	5′-GCTGCCTCTCCCTCGCCTCAGCTGTCTTT-3′
	C	R	5′-AAAGACAGCTGAGGCGAGGGAGAGGCAGC-3′
	G	F	5′-GCTGCCTCTCCCTGGCCTCAGCTGTCTTT-3′
	G	R	5′-AAAGACAGCTGAGGCCAGGGAGAGGCAGC-3′

*orientation of oligo: Forward (F)/Reverse (R) strand.

### Replicated SNP Genotyping in the Norwegian Study Population

DNA for genotyping was extracted from peripheral blood samples stored in the HUNT biobank as described elsewhere [27], [45]. Replicated SNP genotyping was performed at Southwest Foundation for Biomedical Research, Texas, using TaqMan genotyping assays (Applied Biosystems) on an Applied Biosystems' 7900HT Fast Real-Time PCR System. For each TaqMan SNP assay 50 ng of genomic DNA was used in a 5 µl reaction volume with 2.5 µl TaqMan Genotyping master mix, 0.125 µl TaqMan assay mix (40×) and 1.375 µl water. Four no template (water) controls were incorporated into each 384-well plate. SNP genotype clustering and individual sample genotype calls were interrogated using Applied Biosystems' Sequence Detection Systems software v2.2.2.

### Statistical Methods

#### Genotype Error Checking

Genotypes pertaining to the Aust/NZ study population not conforming to Mendelian inheritance laws were identified and assessed using SimWalk2 [50]. Mendelian discrepancies and spurious recombinations were removed by blanking those genotypes identified in SimWalk2 as having a high probability of being in error. Norwegian genotypes in this current study were compared to Norwegian genotypes in our preliminary study using SNPlex technology which prioritized the *TNFSF13B* gene [29].

#### SNP Allele Frequency Estimation

We used the statistical genetics analysis program SOLAR [51] to estimate SNP allele frequencies by using maximum likelihood techniques that account for pedigree structure. Tests for deviations from Hardy-Weinberg equilibrium (HWE) were also performed in SOLAR.

#### SNP Linkage Disequilibrium Estimates

Estimates of pairwise linkage disequilibria parameters were used in a basic correlation method to assess all disequilibria jointly in SOLAR. In this approach, SNP genotypes are scored as -1, 0 and 1 (for the AA, AB and BB genotypes, respectively) and the correlations among these data vectors are calculated to give an unbiased estimate of the squared LD correlation, rho (ρ).

#### SNP Association Analysis

Power calculations and SNP association analyses were performed in SOLAR [51]. SNP association analyses were conducted using SOLAR's QTLD procedure [52]. This procedure performs a test for population stratification and two commonly used association tests: the quantitative transmission disequilibrium test (QTDT) [53], and the measured genotype test [54]. The QTDT procedure is not limited to the scoring of allele transmission from parents to offspring but extends further to assess the entire pedigree structure. The scoring of allele transmission can be performed for quantitative or qualitative traits and it has been modified in SOLAR to work with discrete traits using a threshold model [55]. The measured genotype test uses a standard threshold model assuming an underlying normal distribution of liability. The threshold model and its assumptions are near identical to those used in standard logistic regression but benefits from the ease of interpretation with regard to genetic effects. The measured genotype test of association can assess the extent of genotypic mean differences (or the liability or risk scale) between case and control singletons assuming a model of additive gene action [54]. Due to the non-familial structure of the Norwegian study population, we can only present the measured genotype test statistic for this cohort.

#### Multiple Hypothesis Testing

To accommodate for multiple hypothesis testing, we used the approach of Moskvina and Schmidt [56] to determine the effective number of independent SNPs (i.e. tests) based on the pair-wise genotypic correlations. This algorithmic approach has been implemented into SOLAR and it evaluates the strength of correlation amongst the observed genotypes at each SNP locus within a gene.

## Results

### Statistical Power analyses

We performed formal power calculations to assess the power to detect an association (between a SNP and the dichotomous preeclampsia phenotype – where affected are scored as 1 and unaffected as 0) of a given relative size in the population. In the Aust/NZ families, with a SNP-specific heritability of 0.01 to 0.05, we predicted 80% power to identify functional effects that account for as little as 3.5% of the total phenotypic variation with a nominal alpha (significance) of 0.05. In the Norwegian case/control cohort, we estimated an 80% likelihood of identifying a SNP accounting for at least 2% of the total (dichotomous) phenotypic variation.

### *TNFSF13B* Gene Sequencing

**Figure 1:**
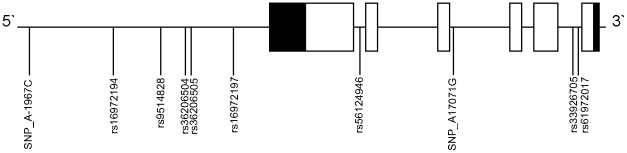
Schematic representation of the *TNFSF13B* gene and variants detected in a sub-set of founding or proband preeclamptic women from the Aust/NZ study population. Solid blocks; untranslated exons, open blocks; translated exons.

The proximal promoter (2 kb upstream of the translation start site), the 5′UTR, 3′UTR and all coding regions were sequenced. In total, we identified nine SNPs (two novel, seven known) and one known, single base insertion in the proximal promoter or intronic sequence flanking the exons (Table 3 and Figure 1).

**Table 3:** *TNFSF13B* variants tested in the Aust/NZ and Norwegian study populations.

SNP	Chr. Post. (bp)*	Function	Aust/NZ study population				Norwegian study population		
			Allele (frequency)		MG_p_	QTDT_p_	Allele (frequency)		MG_p_
SNP_A-1967C	107718278	pp	Failed genotyping assay				Not tested		
rs16972194	107718962	pp	G (0.993)	A (0.007)	0.380	0.015	G (0.997)	A (0.003)	0.421
rs9514828	107719374	pp	G (0.566)	A (0.434)	0.406	0.915	Not tested		
rs36206504	107719569	pp	A (0.965)	C (0.035)	0.714	0.162	Not tested		
rs36206505	107719584	pp	A (0.931)	G (0.069)	0.125	0.618	Not tested		
rs16972197	107719892	pp	G (0.993)	C (0.007)	0.380	0.015	G (0.997)	C (0.003)	0.357
rs56124946	107720644	Intron 1	C (0.993)	G (0.007)	0.380	0.015	C (0.991)	G (0.009)	0.737
SNP_A17071G	107737282	Intron 3	Failed assay design				Not tested		
rs33926705	107757082^∧^107757083	Intron 5	Not tested				Not tested		
rs61972017	107757114	Intron 5	A (0.988)	C (0.012)	1.000	0.197	Not tested		

Novel SNPs are denoted SNP_[UCSC reference template allele][bp position from TSS][alternative allele]. Alleles reported are orientated on the TOP strand (ftp://ftp.ncbi.nih.gov/snp/database/Illumina_top_bot_strand.note.txt). * ref_assembly, human genome build 36.3, Abbreviations: TSS; translation start site, Chr.; chromosome, Post.; position, bp; base pair, MG_p_; measured genotype test p-value, QTDT_p_; quantitative transmission disequilibrium test p-value, pp; proximal promoter.

### *TNFSF13B* Genotyping and Association Analysis in the Aust/NZ Families

The 74 Family Cohort (n = 480) included 140 affected women (20 with eclampsia, 120 with preeclampsia) and 146 unaffected women (normotensive and non-proteinuric). At the time of custom SNP pool design the single base insertion variant (rs33926705) could not be included into the assay. Additionally, one novel SNP (SNP_A17071G) failed assay design due to it residing within a duplicate or repetitive region and the other novel SNP (SNP_A-1967C) could not be successfully genotyped. The seven successfully typed SNPs in the Aust/NZ study population exhibited a high sample genotype success rate (≥98.5%) and all SNPs conformed to Hardy-Weinberg expectations (p>0.05).

**Figure 2:**
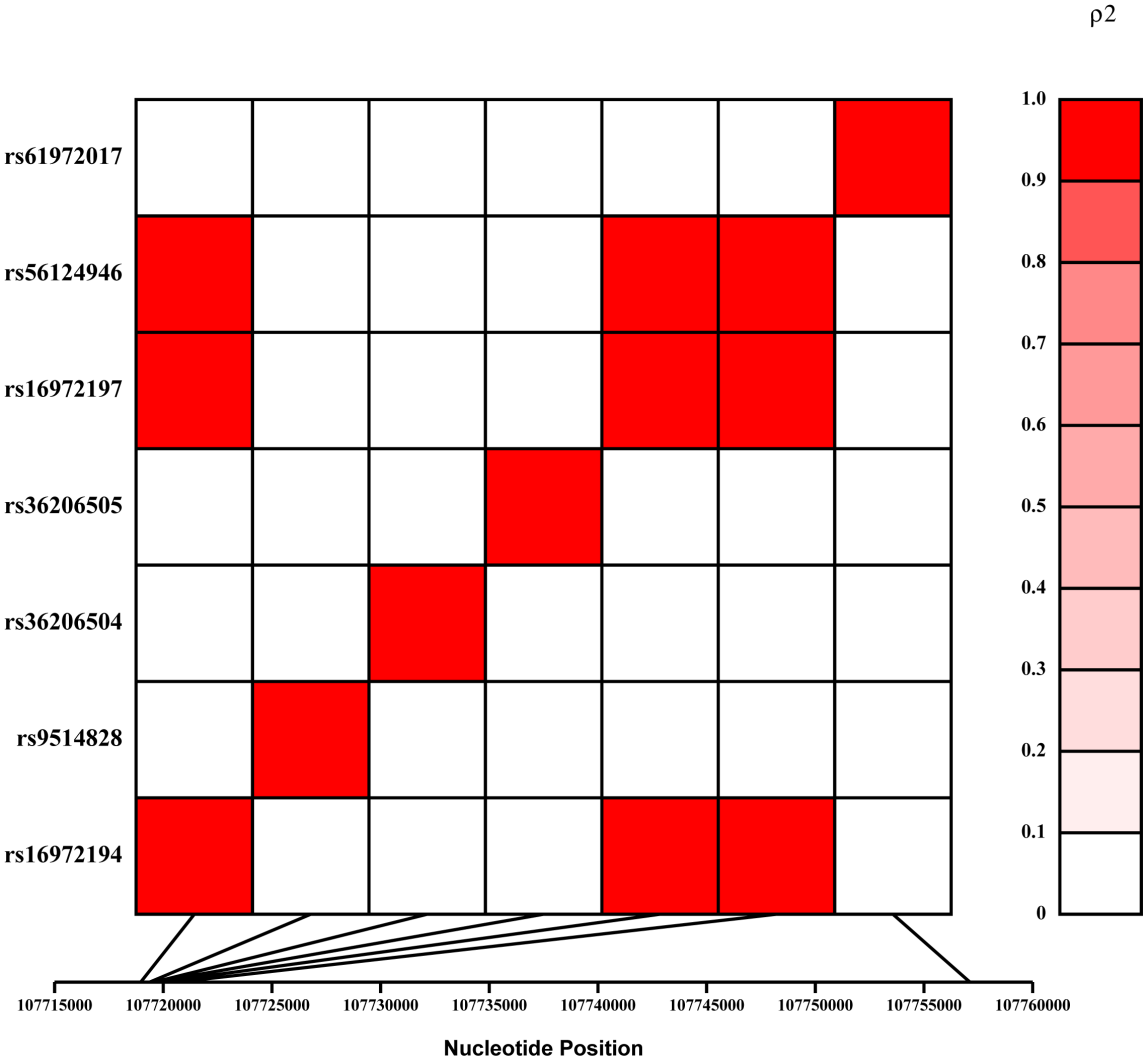
Linkage disequilibrium (LD) pattern for the successfully genotyped *TNFSF13B* SNPs in the Aust/NZ study population. LD is measured by the squared value of the pair wise correlation (rho) amongst intra-genic genotypes and the strength of correlation is depicted in the colored bar to the right of the LD plot. The intensity of red color increases with the strength of SNP allele correlation from white (0) indicating no correlation (i.e. no LD) to red (1.0) indicating a perfect correlation (i.e. complete LD).

We observed association to preeclampsia (p = 0. 0153) for three rare SNPs (rs16972194, rs16972197 and rs56124946) (Table 3). Based on the extent of linkage disequilibrium (LD) between these SNPs (Figure 2) we were effectively testing five independent SNPs in our association analyses. To correct for multiple testing, these SNP correlations return an adjusted p-value threshold of 0.0102. Therefore, we present a borderline association for three *TNFSF13B* SNPs (Figure 2) with preeclampsia susceptibility in the Aust/NZ families with the QTDT statistic (Table 3).

### Bioinformatic Evaluation

Bioinformatic analysis of rs16972194, rs16972197 and rs56124946 using MATCH™ 1.0 [57] revealed that the rare rs16972194 (A) allele created a promoter sequence with high core similarity (core match; 0.948, matrix match; 0.932) to the binding motif of transcription factor Oct-1. Oct-1 is a member of the POU domain transcription factor family [58], and the DNA recognition sequence is the octamer motif 5′-ATGCAAT-3′, which is shared between several Oct/POU transcription factor family members [59]. A more stringent bioinformatics analysis, examining whether any other known transcription factor(s) could bind preferentially to the minor allele, but not the major allele of rs16972194, was also performed. A total of 1,351 binding models for transcription factors were collected from the TRANSFAC (version 2009.2) and JASPAR CORE databases [48], [60]. The Oct- motif was confirmed, and additional shorter core sequences exhibiting a preference to the rs16972194 minor (A) allele were identified. Of these, the FOXC1 and YY1 transcription factor-motifs were the most relevant.

### Electrophoretic Mobility Shift Assays (EMSA)

**Figure 3:**
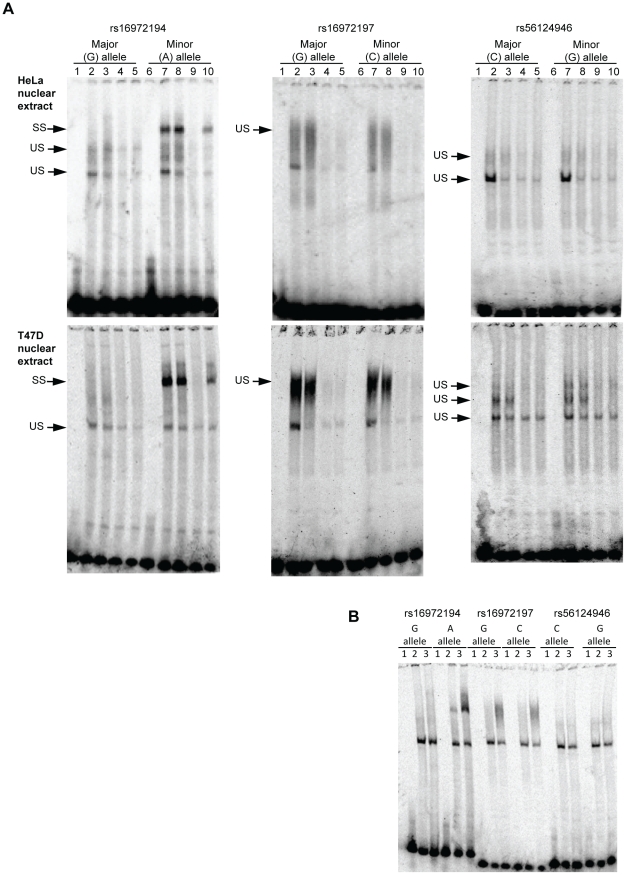
Electrophoretic mobility shift assays for the *TNFSF13B* SNPs associated with preeclampsia in the Aust/NZ families. Panel A: Lanes 1 and 6; No nuclear extract, Lanes 2 and 7; nuclear extract only, Lanes 3 and 8, Nuclear extract with unspecific competitor, Lanes 4 and 10; Nuclear extract with specific competitor (unlabelled double stranded oligo for the major allele), Lanes 5 and 9; Nuclear extract with specific competitor (unlabelled double stranded oligo for the minor allele). Panel B: Major shifts without competitor. Lane 1; no nuclear extract, Lane 2; HeLa nuclear extract, Lane 3; T47D nuclear extract. SS; specific shift, US; unspecific shift.

We subsequently carried out electrophoretic mobility shift assays (EMSA), using nuclear extracts from HeLa and T47D cells. Radioactively labeled double stranded DNA oligonucleotide probes representing the major and minor allele of each of the three rare and associated SNPs were run with both nuclear extracts to visualize binding of nuclear protein (Figure 3). All probes demonstrated non-specific electrophoretic mobility shifts (Figure 3). Unlabelled double stranded oligos for the wild type and mutant alleles, as well as an unspecific competitor, were added in separate reactions. The unspecific shifts were inhibited by these competitors. Interestingly, the rs16972194 SNP demonstrated a specific shift for the minor, but not the major, allele probe (Figure 3). The minor allele unlabelled probe suppressed the shift whereas the major allele unlabelled probe and the unspecific competitor did not (Figure 3). This strongly suggests the creation of a nuclear factor binding site by this variant. Antibodies for transcription factors Oct1, Oct2, Oct3/4, Oct6, YY1 and FOXC1 were run in separate reactions, but no supershift was observed under the current running conditions (result not shown).

### Replicated *TNFSF13B* SNP Genotyping and Association Analysis in the Norwegian Singletons

DNA samples were available for 851 confirmed cases of women with preeclampsia and 1,440 women with a history of non-preeclamptic pregnancies (controls). Of the available cases, 737 women were registered with one and 114 women with more than one preeclamptic pregnancy. As expected, gestational age (273 d vs. 282 d, p<0.001) and birth weight (3156 g vs. 3457 g, p<0.001) differed between the neonates in preeclamptic and non-preeclamptic pregnancies. Maternal age at first pregnancy was higher in the case group (23.6 yrs vs. 22.8 yrs, p<0.001), but the groups did not differ with respect to parity (2.56 vs. 2.55, p>0.05). After adjusting for maternal age, the differences in clinical phenotype between case and control groups remained significant (p<0.001). All three rare SNPs associated to preeclampsia were successfully genotyped in the Norwegian study population. A high genotyping success rate (≥97.1%) was observed and all three SNPs were in Hardy-Weinberg equilibrium (p>0.05). Independent genotyping of these SNPs did not replicate the results attained in the Aust/NZ families (Table 3).

## Discussion

The elucidation of genetic risk factors contributing to preeclampsia susceptibility has become a priority of obstetric research. It is well known that both maternal and paternal factors influence the preeclampsia phenotype [9], [11], [13], [14]. To identify maternal genetic contributions to preeclampsia our positional cloning approach identified a susceptibility QTL on chromosome 13q [20], and the *TNFSF13B* gene was prioritized as the most promising candidate under this QTL [29]. In the current study, a targeted molecular genetic evaluation of TNFSF13B was undertaken. We report borderline association to a putative functional SNP within the proximal promoter region of *TNFSF13B* with preeclampsia susceptibility in affected Aust/NZ families. The finding is not replicated in a Norwegian case/control population cohort.

The early changes of pregnancy include a shift of the Th1/Th2 cytokine balance towards Th2 predominance [61]. Inflammatory/infectious processes may alter this balance towards a Th1 profile less favorable for pregnancy [61]. TNFSF13B is regulated by inflammatory response cytokines [62]–[64] and stimulates macrophages to secrete proinflammatory cytokines, enhancing the cascade [32]. Interference with the homeostatic regulation of *TNFSF13B* could therefore potentially disturb the finely tuned cytokine balance of pregnancy. Decidual stromal cells (DSCs) have been shown to express *TNFSF13B* mRNA and protein [40] and DSCs are involved in a number of different functions that are important for the immunological cross-talk between mother and fetus [65]. Our finding may therefore reflect an abnormal immunological function of DSCs at the maternal-fetal interface.

The interaction between decidual natural killer (NK) cells and the allogenic extravillous trophoblast (EVT) cells is suggested to contribute to the depth of EVT cell invasion during implantation and placentation [66], [67]. NK-cells are the predominant leucocytes found in decidua [67] and NK- cell activity is elevated by TNFSF13B in mice [68], [69]. In humans, TNFSF13B has been shown to relay immunological response to toll-like receptor (TLR) 3 and 4 binding [70], [71]. TLRs are expressed on placental NK cells. They help discriminate between “self” and “non-self”, and have been shown to recognize infectious agents as well as endogenous danger signals [61], [72]. These biological functions are implicated in preeclampsia pathogenesis [5], [10], [73], and TLRs have been assigned a role in pregnancy-associated complications such as intrauterine growth restriction, pre-term delivery and preeclampsia [74]. It is therefore tempting to speculate, that disturbed TLR signaling might be one mechanism by which aberrant *TNFSF13B* regulation could confer susceptibility to preeclampsia.

We observe differential nuclear binding to the minor allele of the *TNFSF13B* promoter area rs16972194 SNP, thus suggesting it as a putative functional, albeit rare, variant. A recent report showed that SNPs contribute substantially to genetic variation leading to aberrant transcription factor binding, and that this might be an important evolutionary mechanism [75]. Transcriptional regulation is proving to be highly complex, as illustrated by the FANTOM consortiums attempt to describe the transcriptional landscape of the mouse genome [76]. In the human genome, over 2,500 proteins with DNA binding motifs are predicted, and it is estimated that about 8% of human proteins are transcription factors [77]. Of these, only about 10% are well characterized and included in available databases for motif searches [48], [60]. HeLa cells are widely used as a model system for biomedical research on both normal and disease molecular processes [78]. A wide variety of nuclear factors are expressed in this cell type, including transcription factors only expressed in embryonic stem-cells and not in differentiated tissues [79]. Our EMSA results show differential binding of a nuclear factor to the sequence in question. The finding was replicated using T47 cells. However, further investigation of the protein band representing nuclear factor binding to the rs16972194 minor allele and in vivo confirmation of the result is warranted. The role of the identified putative functional variant in other TNFSF13B related diseases should also be subject of further investigation.

The Aust/NZ sequencing sample set ensures a high probability of detecting common frequency variants within the population. However, our choice of affected women who are either pedigree founders or probands for re-sequencing will also increase the likelihood of identifying rare functional variants that are enriched in these preeclamptic women. Over the last decade, a large number of genome wide association studies have been undertaken for numerous common complex diseases, assuming that common disease is caused by common variation (the common disease-common variant (CDCV) hypothesis) [80]. This approach has provided new insight [81], but a notable knowledge “gap” of 90–95% of the genetic liability to these diseases is left unaccounted for [80]. As shown for extensively studied disease genes, such as *BRCA1* and *BRCA2*, rare variants might be population specific, but yield a higher individual risk of disease than common variants (the common disease rare-variant (CDRV) hypothesis) [82], [83]. Therefore, most geneticists appreciate that the CDCV and CDRV hypotheses both have their place in the understanding of heterogeneous genetic disorders. The rare *TNFSF13B* variants exhibiting borderline association with preeclampsia susceptibility in the Aust/NZ families were not replicated in the Norwegian population sample. Confirming the biological importance of rare predisposing variants between populations is a challenge [80], and further genetic and molecular investigation in other populations is required.

The Norwegian population cohort has a larger sample size than the Aust/NZ family cohort. However, the power of a study is also influenced by the stringency of the diagnosis and the pedigree information included in the statistical analyses. In both the Aust/NZ and Norwegian study populations, the preeclampsia diagnosis was based on the development of new onset hypertension and proteinuria during pregnancy. However, preeclampsia is a complex disease, and preeclamptic cases selected from a population sample represent a more heterogeneous group, than a collection of family samples. The MBRN did not include absolute values of blood pressure and proteinuria, and severity of preeclampsia was not reported to the registry before 1998. Thus, we are not able to include this information in our analyses. However, women with a familial disposition generally display more severe manifestations of the disease [84], and this may have influenced our results. The available information about relatedness in the Aust/NZ pedigree sample set allows a wider range of potential test statistics to be considered. Hence, we applied both the measured genotype association test and the QTDT which controls for any potential latent stratification in the data. In the absence of hidden stratification and residual linkage effects, the measured genotype test is asymptotically more powerful than the QTDT [85]. However, in the presence of certain types of latent stratification, the QTDT can be more powerful. Similarly, residual linkage (reflective of additional functional variants near the associated marker) can also lead to a more powerful QTDT. Such additional potential genetic signals have no influence in the analysis of unrelated individuals. Thus, even though the sample size of the Norwegian cohort is larger its composition of solely unrelated females may have rendered it somewhat less powerful for detecting the observed effect if such complexities are involved.

In conclusion, we observe borderline association between three rare *TNFSF13B* SNPs, one of which exhibits putative functional characteristics, and maternal preeclampsia genetic susceptibility in our Aust/NZ families. Our observation supports *TNFSF13B* as a potential preeclampsia susceptibility gene in a region of known genetic linkage, and adds evidence to its importance for a successful human pregnancy. Furthermore, showing differential nuclear factor binding to the minor allele of rs16972194, we propose this variant as a candidate for additional functional evaluation.
